# Supplementary material for: Percolation transition of K-destinations reveals universal backbone of urban mobility networks
Source: R Soc Open Sci. 2026 Sep 9;13(9):251865. doi: 10.1098/rsos.251865 (PMC13561036; doi:10.1098/rsos.251865)
Supplement: Supplementary file 1 [file rsos.251865.zip › rsos.251865/rsos251865_si_001.pdf]

Supplementary Materials for Percolation Transition of K-destinations Reveals Universal Backbone of Urban Mobility Networks

Weiyu Zhang,[1] Furong Jia,[1] Jianying Wang,[2] Yu Liu,[1] and Gezhi Xiu[1]

[1] *Institute of Remote Sensing and Geographical Information Systems, Peking University, Yiheyuan Road 5, Haidian District, Beijing, 100871, China*

[2] *Thrust of Urban Governance and Design, Society Hub, The Hong Kong University of Science and Technology - Guangzhou Campus, No. 1 Duxue Road, Nansha District, Guangzhou, Guangdong, 511453, China*

CONTENTS

Data Description 1
- Mobile Phone Data 1
- Socioeconomic Factors 2

Implementation Details of the Percolation Model 3

Network Topological Properties 3
- Authority Analysis 3
- Clustering Coefficient Calculation 4
- Integration Process of Low-in-degree Nodes 4
- Two Stage Densification of Hubs 4

Implementation Details of Synthetic Networks 5
- Random Graphs 5
- Randomized Percolation Process 5
- Gravity Model 6

Community Detection 6

Power-law Exponent Estimation 6
- Spatial Autocorrelation of Added Destinations across Rank Ranges 7

Spatial distribution of the Proportion of Principal Destinations 7
- Los Angeles 7
- Philadelphia 7

Relationship between socioeconomic factors and PPD 8
- Correlation of socioeconomic factors with PPD 8
- Shift of the significance around the connectivity threshold 8

References 15

DATA DESCRIPTION

Mobile Phone Data

We utilize a comprehensive dataset detailing the intracity human mobility within the United States, spanning eight major cities from January 2018 to December 2021[1]. This dataset records mobility patterns within the administrative boundaries at the city level, employing the Census Block Group (CBG) as the spatial unit—a statistical area used in the United States for conducting population and housing censuses. Movements are compiled into monthly Origin-Destination (OD) matrices. In each matrix, the flow from one CBG to another quantifies the total visits made by

residents who lived in the origin CBG to the destination within the month. It should be noted that, despite revisions to the CBG in 2020, this dataset consistently uses the 2010-2019 version to ensure data uniformity.

The cities included are New York, Los Angeles, Chicago, Phoenix, Philadelphia, San Diego, Dallas, and San Jose. The CBG count, population and average monthly flow volume of each city are shown in Table S1. These cities vary a lot in their size, shape, history, and socio-economic state, ranging from San Jose with 583 CBGs to New York with 6,493 CBGs. The average monthly flow volume varies from 23 million in San Jose to almost 220 million in New York, depending on their city scales. This heterogeneity emphasizes the dataset's potential to delineate diverse dimensions of urban connectivity, consequently bolstering the generalizability of our study's findings.

TABLE S1: Statistical summary of the eight U.S. cities.

**City**	**CBG count**	**Population (millions)**	**Avg. monthly flow (millions)**
New York, NY	6,493	8.08	219.61
Los Angeles, CA	2,509	3.78	92.16
Chicago, IL	2,183	2.55	49.09
Philadelphia, PA	1,336	1.49	33.83
Phoenix, AZ	972	1.70	41.13
Dallas, TX	946	1.37	25.84
San Diego, CA	845	1.42	38.54
San Jose, CA	583	1.02	22.96

Socioeconomic Factors

The socioeconomic dataset, which is part of [2], is obtained from the American Community Survey (ACS) and OpenStreetMap, and utilizes CBG-level information. This dataset encompasses key indicators including poverty, health insurance coverage, educational levels, the built environment, and racial segregation (Table S2), spanning from 2018 to 2021 and covers eight major U.S. cities, including New York, Los Angeles, Chicago, Phoenix, Philadelphia, San Diego, Dallas, and San Jose.

TABLE S2: Socioeconomic Indicators

**Type**	**Indicator**
Poverty	Median Household Income
	The Ratio Of Income To Poverty Level Between 0.5 And 0.99
	The Ratio Of Income To Poverty Level Below 0.5
Health	The Ratio Of Population With No Health Insurance
Education	Average Population Enrolled In College
	Average Population That Graduated From High School
	Average Population With A Bachelor's Degree
	Average Population With A Master's Degree
	Average Population With A Doctorate
Built Environment	Building Density
	Driving Road Density
	Cycling Road Density
	Walking Road Density
	POI Density
	Construction Percentage
	Residential Percentage
	Racial Entropy

IMPLEMENTATION DETAILS OF THE PERCOLATION MODEL

To determine the integration threshold for each city-month pair, the process begins with setting $K = 1$. We incrementally increase K by one, and each time select the top K outflows from each node to form a ‘top-K network’. This process continues until the top-K network achieves strong connectivity, meaning every node can be reached from any other node. The value of K at this point is identified as the integration threshold for the city-month pair.

In our analysis, we observed a saturation phenomenon in the integration threshold as we progressively removed the least important nodes, i.e., those with the smallest degrees in the city. Specifically, as more nodes with the lowest degrees are removed, the integration threshold initially decreases and then stabilizes. This indicates that the least significant, peripheral areas disrupt the calculation of connectivity, while the core, stable urban areas’ critical connectivity remains largely unaffected by the removal of additional nodes. The behaviour of this core urban area is precisely what we aim to measure and depict. To illustrate this phenomenon, we present the distribution of integration thresholds after removing 2%, 3%, and 5% of nodes with the smallest degrees in Figure S1. The results show no significant changes as more of the least connected nodes are removed, underscoring the robust structure of core urban areas. In our principal findings, we opted to exclude the bottom 2% of nodes.

TABLE S3: Summary statistics of monthly integration thresholds across eight U.S. cities.

City	Mean	Median	Std. dev.	City	Mean	Median	Std. dev.
New York, NY	173.92	170.5	48.15	Philadelphia, PA	124.69	123.5	36.36
Los Angeles, CA	152.08	131.5	64.85	San Diego, CA	163.90	142.0	54.81
Chicago, IL	122.38	115.5	43.69	Dallas, TX	133.56	132.5	31.71
Phoenix, AZ	146.48	140.5	36.06	San Jose, CA	119.38	118.0	24.79

NETWORK TOPOLOGICAL PROPERTIES

Authority Analysis

To identify major urban destinations and track their emergence across different connectivity levels, we calculated authority scores for nodes in the K-most-frequent-destination networks using the Hyperlink-Induced Topic Search (HITS) algorithm [3].

The HITS algorithm computes two complementary scores for each node in a directed graph: authority scores and hub scores. The authority score $a(v)$ measures how important node v is as a destination (receiving flows), while the hub score $h(v)$ measures how important node v is as an origin (sending flows). These scores are computed iteratively:

$$a(v) = \sum_{u:(u,v)\in E} h(u) \cdot w_{uv}, \quad h(v) = \sum_{u:(v,u)\in E} a(u) \cdot w_{vu}, \tag{1}$$

where E is the edge set and w_{uv} is the weight of the edge from node u to node v. The algorithm starts with uniform initial values and updates the scores iteratively with L2 normalization until convergence.

For our analysis, we focused on the authority scores, which indicate the importance of nodes as destinations in the urban mobility network. For each network G_k, we identified the top 10 nodes with the highest authority scores and calculated their mean:

$$H_k = \frac{1}{10} \sum_{i=1}^{10} a_i^{(k)}, \tag{2}$$

where $a_i^{(k)}$ represents the authority score of the i-th highest-ranking node in the K-most-frequent-destination network G_k. This metric, referred to as “top authority” in the main text, tracks how the concentration of flows to major destinations changes as the network expands. All calculations were performed using the NetworkX library implementation of the HITS algorithm.

Clustering Coefficient Calculation

We computed the average clustering coefficient to characterize the degree of local closure within the top-K mobility networks. This metric quantifies the tendency of nodes to form locally connected triplets, providing a summary of triangle closure in the network.

For each directed top-K network G_K, we first identified its giant strongly connected component (GSCC) and then converted this component to its undirected counterpart. For each node i in the resulting undirected graph, the local clustering coefficient was defined as

$$CC_i = \frac{2T_i}{k_i(k_i - 1)}, \tag{3}$$

where T_i is the number of triangles passing through node i, and k_i is the degree of node i in the undirected version of the GSCC of G_K. For nodes with degree less than 2, CC_i was set to 0.

The network-level clustering coefficient was then computed as the average over all nodes:

$$\overline{CC}_K = \frac{1}{N_K} \sum_{i=1}^{N_K} CC_i, \tag{4}$$

where N_K is the number of nodes in the undirected version of the GSCC of G_K.

Integration Process of Low-in-degree Nodes

To complement the main-text analysis of the integration threshold, we examined whether the observed threshold represents a robust network-scale property, rather than being set mainly by a small number of exceptionally weakly reached nodes. We tracked the fraction of nodes with in-degree $= 0$, in-degree ≤ 1, and in-degree ≤ 2 as a function of K in the top- K mobility networks. These quantities measure how many locations remain only weakly reached by the rest of the city as additional destinations are retained. The statistics were computed for each city-month network after the same preprocessing and top-K construction used in the main analysis.

Across all cities, the fractions of low-in-degree nodes decrease monotonically with K and approach zero within a relatively narrow rank regime near the empirical integration scale (Figure S2). Importantly, this pattern is observed not only for in-degree $= 0$, but also for the more permissive thresholds in-degree ≤ 1 and in-degree ≤ 2, indicating that weakly reached nodes are absorbed collectively rather than being eliminated only at the extreme tail.

This result suggests that the integration threshold near the representative scale of $K \approx 130$ is not determined by a few outliers. Instead, it reflects a robust, system-wide incorporation of weakly connected locations into the city-wide mobility structure, reinforcing the interpretation that the integration regime captures a genuine structural scale rather than an artifact of a few peripheral cases.

Two Stage Densification of Hubs

To further support the main-text interpretation that the steeper in-degree distribution at K^*, relative to $K^*/2$, is associated with the inclusion of destinations that are important but not limited to the most dominant hubs, we examined how the maximum in-degree within each strongly connected component (SCC) changes as additional destinations are retained. For each top-K network, we computed the in-degree of every node, identified the maximum in-degree within each SCC, and then averaged this quantity across all SCCs. This statistic captures the typical concentration of incoming links around the most connected node within a component.

As shown in Figure S3, across cities, this quantity exhibits a two-stage growth pattern. From $K = 1$ up to approximately $K^*/2$, it increases rapidly, indicating that the earliest retained destinations mainly reinforce the most connected nodes within components. In this regime, the internal structure of SCCs becomes increasingly concentrated around a limited number of highly connected destinations.

From $K^*/2$ to K^*, the mean maximum in-degree continues to increase, but at a slower rate. This suggests that destinations added in this rank range remain structurally important, while their contribution is no longer limited to further amplifying only the already most connected nodes. Instead, this pattern is consistent with an expansion of the set of important destinations across components.

This two-stage pattern provides additional support for the interpretation that the steepening of the in-degree distribution from $K^*/2$ to K^* may reflect newly added destinations that are no longer concentrated only among the strongest hubs, but extend to a broader set of lower- and intermediate-popularity destinations.

IMPLEMENTATION DETAILS OF SYNTHETIC NETWORKS

To demonstrate that the empirical city-month integration thresholds, which concentrate around a representative scale near 130, emerge from inherent urban organizational properties rather than basic network connectivity patterns, we implemented a set of synthetic network models with varying degrees of structural complexity. Each model preserves selected structural characteristics while randomizing others, enabling isolation of specific mechanisms underlying the observed percolation behaviour.

To compare with the empirical threshold distribution calculated from 48 monthly networks per city, for each synthetic model, we synthesized a counterpart network for each city-month network with the same scale, $|V_{\text{synthetic}}| = |V_{\text{empirical}}|$. This specification yields 48 synthetic networks for each synthetic model per city. Threshold distributions are then estimated using identical procedures with empirical networks, ensuring that observed differences reflect genuine structural distinctions rather than methodological artifacts or sampling biases.

Random Graphs

Three random network models are implemented to synthesize networks with topological properties that are increasingly aligned with those of the empirical networks. The first model is a directed Erdős–Rényi variant where each node randomly selects destinations with uniform connection probability. Each potential edge (i, j) exists with probability $p = m/[n(n-1)]$, where m represents the total number of edges in the empirical network and n is the node count. This model serves as the baseline random network, preserving only the overall edge density while completely randomizing the connection patterns.

The second model introduces node heterogeneity by preserving the out-degree sequence of empirical networks. Each synthetic node i corresponds to a specific node in the empirical network and maintains the same out-degree: $d^{\text{out}}_{i,synthetic} = d^{\text{out}}_{i,empirical}$. The connection probability from node i to any target node j is calculated as:

$$p_{i\to j} = \frac{d_i^{\text{out}}}{n-1},$$

In this model, target selection remains random, resulting in a randomized in-degree distribution that typically follows a Poisson-like pattern.

The third model simultaneously preserves both in-degree and out-degree sequences of empirical networks. The probability of establishing a directed edge from node i to node j is calculated as:

$$p_{i\to j} = c \cdot \frac{d_i^{\text{out}} \cdot d_j^{\text{in}}}{(n-1)^2},$$

where d_i^{out} and d_j^{in} represent the empirical out-degree and in-degree respectively, and c is a normalization constant that ensures the expected total number of edges matches the empirical network. This configuration model preserves the complete degree sequence while randomizing the specific edge assignments based on degree compatibility.

For all three models, edge weights were assigned by sampling from the weight distribution of empirical networks.

Randomized Percolation Process

The randomized integration threshold process tests the functional irreplaceability of high-volume connections by substituting deterministic ranking with probabilistic selection in the percolation process. For each node i, we denote $\mathbf{f}_i = (f_i^1, f_i^2, \ldots, f_i^N)^T$ as the empirical flow vector containing outflow volumes to all N nodes in the network. At each iteration $k = 1, 2, 3, \ldots$, we select an unchosen destination node j for each node i with probability proportional to the remaining elements of $\mathbf{f}_i$:

$$P_{ij} = \frac{f_i^j}{\sum_{l\in R_i} f_i^l},$$

where P_{ij} represents the probability of selecting destination node j from source node i, f_i^j is the empirical flow volume from node i to node j, and R_i denotes the set of remaining unselected destination nodes for source node i. The iteration process continues until the resulting K-most-frequent-destination network achieves strong connectivity, at which point the algorithm terminates and records the connectivity threshold K^*. This approach enables comparison between probabilistic selection and the deterministic ranking approach used in empirical networks.

Gravity Model

The gravity model incorporates spatial distance decay effects to test whether geographic proximity can account for the observed integration threshold, contrasting with empirical networks that reflect both spatial and functional relationships. For each pair of Census Block Groups, distances were calculated as great-circle distances between their centroids using the Haversine formula. Following the widely accepted gravity model formulation [4], the synthetic flow weight between nodes i and j is calculated as:

$$w_{ij}^{\text{gravity}} = c \cdot \frac{F_i^{\text{in}} \cdot F_j^{\text{out}}}{d_{ij}^{\beta}},$$

where F_i^{in} and F_j^{out} represent the total empirical inflow and outflow respectively, d_{ij} is the great-circle distance between centroids, β is the distance decay exponent controlling strengths of distance decay, and c is a normalization constant.

The distance decay parameter β was optimized for each city by testing values from 0.5 to 3.0 in increments of 0.1, selecting the value that minimized root mean square error between predicted and observed flows. The normalization constant c was calculated to preserve total flow volume:

$$c = \sum_{i,j} f_{ij}^{\text{empirical}} / \sum_{i,j} \frac{F_i^{\text{in}} \cdot F_j^{\text{out}}}{d_{ij}^{\beta}}.$$

COMMUNITY DETECTION

Community detection analysis employed the Asynchronous Fluid Communities algorithm [5] to identify functionally coherent mobility regions within both empirical and gravity model networks. We converted directed mobility networks to undirected ones by symmetrizing adjacency matrices through $A'(i,j) = A(i,j) + A(j,i)$. The algorithm iteratively assigns nodes to communities based on neighbor label frequencies, with convergence achieved when community assignments stabilize (less than 1% change between iterations). The number of communities was set to 50 without loss of generality, yielding community sizes averaging 27 CBGs that align with established urban planning scales such as neighborhood districts or traffic analysis zones. This scale captures sufficient CBGs to reflect functional complementarity among different land uses within each community, while avoiding excessive aggregation that would make spatial heterogeneity in mobility patterns undetectable.

To compare the subtle differences induced by distance-based mobility patterns versus empirical mobility patterns in urban spatial organization, we selected the most similar community between the empirical network and the gravity model network. The similarity between communities is measured by F1 scores, which were calculated as

$$F_1 = 2 \cdot \frac{\text{precision} \cdot \text{recall}}{\text{precision} + \text{recall}},$$

where precision represents the fraction of CBGs in the gravity model community that belong to the corresponding empirical community, and recall represents the fraction of empirical community CBGs captured by the gravity model community. The most similar community pairing was determined via the largest F1 score. This analysis revealed systematic organizational differences: gravity model communities primarily reflected geographical proximity, while empirical communities additionally captured functional complementarities such as the co-clustering of Philadelphia International Airport with downtown areas despite substantial physical separation.

POWER-LAW EXPONENT ESTIMATION

To rigorously estimate the power-law exponent of most-frequent-destination networks, we employed maximum likelihood estimation (MLE) following the methodology introduced by Clauset et al. [6]. This approach addresses the well-documented limitations of linear regression on log-transformed data, which can introduce systematic biases in parameter estimation and lead to spurious identification of power-law behaviour.

For each network's in-degree sequence $\{d_1, d_2, \ldots, d_n\}$, we first determined the optimal lower bound $d_{\min}$ by minimizing the Kolmogorov-Smirnov (KS) statistic between the empirical distribution and the fitted power-law model

across all possible values of $d_{\min}$. The power-law exponent α was then estimated using MLE:

$$\hat{\alpha} = 1 + n \left[\sum_{i=1}^{n} \ln \frac{d_i}{d_{\min} - 0.5} \right]^{-1}$$

where n is the number of degree observations satisfying $d_i \geq d_{\min}$, and the -0.5 correction accounts for the discrete nature of degree distributions. All power-law analyses were implemented using the powerlaw Python package [7], with convergence criteria of 10^{-8} for iterative MLE procedures.

Spatial Autocorrelation of Added Destinations across Rank Ranges

In the main text, we propose a plausible interpretation of the negligible slope change beyond K^*: destinations added beyond K^* are less strongly organized and more noise-like than those added between $K^*/2$ and K^*. To further support this interpretation, we examined whether destinations added in these two rank ranges differ in their spatial autocorrelation. The underlying idea is straightforward: if newly added destinations remain concentrated around important functional destinations, they should show stronger spatial autocorrelation, as the spatial resolution of a CBG is typically smaller than the spatial extent of a functional hub. By contrast, if destinations added are more noise-like, their spatial distribution should be more diffuse and exhibit weaker spatial autocorrelation.

For each city-month network, we divided the added destinations into two rank ranges: $K^*/2 \to K^*$ and $K^* \to 2K^*$. For each range, we counted, for every node, how many times it was selected as a destination, and then computed Moran's I on these destination frequencies. The spatial weight matrix used to compute Moran's I was constructed using Queen contiguity, such that two CBGs were treated as neighbors if their polygons shared either a boundary segment or a vertex. A higher Moran's I indicates stronger spatial autocorrelation of destination frequencies. For each city, we then summarized the mean Moran's I for each rank range across months, together with 95% confidence intervals computed as ± 1.96 standard errors.

As shown in Figure S4, across all cities, the mean Moran's I for destinations in the range $K^*/2 \to K^*$ is higher than that for destinations in the range $K^* \to 2K^*$. Moreover, this difference is statistically significant in seven of the eight cities, with Chicago being the only exception. These results indicate that destinations added between $K^*/2$ and K^* remain more spatially clustered, whereas destinations added beyond K^* are more spatially dispersed.

The lower Moran's I values observed above K^* partially support the interpretation drawn from the slope comparison that destinations added above K^* are spatially less organized than those added between $K^*/2$ and K^*. This pattern is consistent with a gradual shift from stronger preferential organization toward more randomized destination addition as K increases through the percolation process. This comparison provides further microscopic evidence for why K^* is informative beyond its role as the integration threshold.

SPATIAL DISTRIBUTION OF THE PROPORTION OF PRINCIPAL DESTINATIONS

Los Angeles

Figure S5 demonstrates the spatial distribution of the Proportion of Principal Destinations (PPD) across Los Angeles, revealing distinct patterns of urban engagement and socioeconomic status. High PPD values are predominantly associated with affluent neighborhoods, such as Los Feliz, a celebrity-filled enclave adjacent to Hollywood. This proximity to the urban hub of activity reflects its high accessibility and convenience, facilitating easy access to daily services and fulfilling the needs of its residents. Conversely, regions marked by lower PPD values denote medium- to low-income communities, frequently tarnished by crime and insecurity. In particular, neighborhoods around the University of Southern California are notorious for elevated security risks, including widespread gang presence. Areas such as Jefferson Park and Mid City serve as prime examples where diminished PPD values align with socioeconomic challenges and safety issues, adversely affecting their overall habitability and security. In conclusion, the contrast between the high PPD values in Los Angeles' affluent neighborhoods and the lower PPD values in areas fraught with challenges underscores the diverse socioeconomic landscape across the city.

Philadelphia

Figure S6 demonstrates the spatial distribution of PPD values within Philadelphia. Areas with low PPD values are predominantly concentrated in the Northeast and Northern Philadelphia. In certain regions with low PPD

values, a pattern emerges showing proximity to suburban expanses, parks, and rivers, often characteristic of near-suburban residential zones. In addition, areas characterized by low PPD values are commonly associated with poor neighborhoods. One such area is the Lawncrest neighborhood of Philadelphia, which has seen a staggering 291% increase in impoverished residents from 1990 to 2017, indicative of the regional decline. High crime rates often accompany economic hardship in areas like Frankford, which is also marked by a low median household income. Similarly, neighborhoods such as Strawberry Mansion and Hunting Park, situated in the north, are recognized as dangerous zones within Philadelphia, historically infamous for their adverse conditions. Those regions identified by low PPD values, frequently beset with socioeconomic challenges, underscore the intertwined issues of safety and poverty.

RELATIONSHIP BETWEEN SOCIOECONOMIC FACTORS AND PPD

Correlation of socioeconomic factors with PPD

In Fig. 6B, we illustrate the correlation coefficients between seventeen socioeconomic factors and the PPD values across eight cities. Table S4 details the results of the correlation analysis and linear regression of these factors with PPD in New York for June 2018.

TABLE S4: Correlation analysis and linear regression of socioeconomic factors with the Proportion of Principal Destinations (PPD) in New York, June 2018.

**Socioeconomic Factor**	**Slope**	**Intercept**	**R^2**	**p-value**	**Correlation Coefficient**
Median Household Income	0.05	0.95	0.06	$< 1e-3$	0.25
The Ratio Of Income To Poverty Level Between 0.5 And 0.99	−0.04	0.99	0.04	$< 1e-3$	−0.21
The Ratio Of Income To Poverty Level Below 0.5	−0.04	0.99	0.04	$< 1e-3$	−0.20
The Ratio of No Health Insurance	−0.03	0.98	0.02	$< 1e-3$	−0.16
Average Population Enrolled In College	−0.02	0.98	0.02	$< 1e-3$	−0.13
Average Population That Graduated From High School	−0.02	0.98	0.02	$< 1e-3$	−0.13
Average Population With A Bachelor's Degree	0.03	0.95	0.03	$< 1e-3$	0.16
Average Population With A Master's Degree	0.04	0.95	0.04	$< 1e-3$	0.20
Average Population With A Doctorate	0.02	0.96	0.01	$< 1e-3$	0.12
Building Density	0.02	0.96	0.01	$< 1e-3$	0.12
Driving Road Density	0.02	0.96	0.02	$< 1e-3$	0.13
Cycling Road Density	0.03	0.96	0.02	$< 1e-3$	0.14
Walking Road Density	0.02	0.96	0.02	$< 1e-3$	0.12
POI Density	0.01	0.96	0.00	$< 1e-3$	0.05
Construction Percentage	−0.04	0.99	0.00	$< 1e-3$	−0.06
Residential Percentage	−0.02	0.98	0.00	$< 1e-3$	−0.06
Racial Entropy	−0.01	0.98	0.00	$< 1e-3$	−0.07

Shift of the significance around the connectivity threshold

Figure S7 displays the relationship between the number of principal destinations and the p-values of regressions linking socioeconomic factors with the PPD across seven cities, complementing Fig. 6A in the main text. In cities like Chicago and Philadelphia, there is a sharp increase in the significance level of socioeconomic factors, such as racial entropy and driving road density, as the K-most-frequent-destination subnetworks evolve from fragmented components to city-wide connectivity at the integration threshold. By contrast, for subnetworks with $K > K^*$, the significance of many socioeconomic factors becomes weaker and more variable. In cities such as Chicago and San Jose, several factors, including the average population with a doctorate, show an immediate increase in p values once K exceeds K^*. This pattern suggests that associations between PPD and socioeconomic factors are often strongest around the integration threshold, while becoming less stable as additional destinations are included beyond this regime.

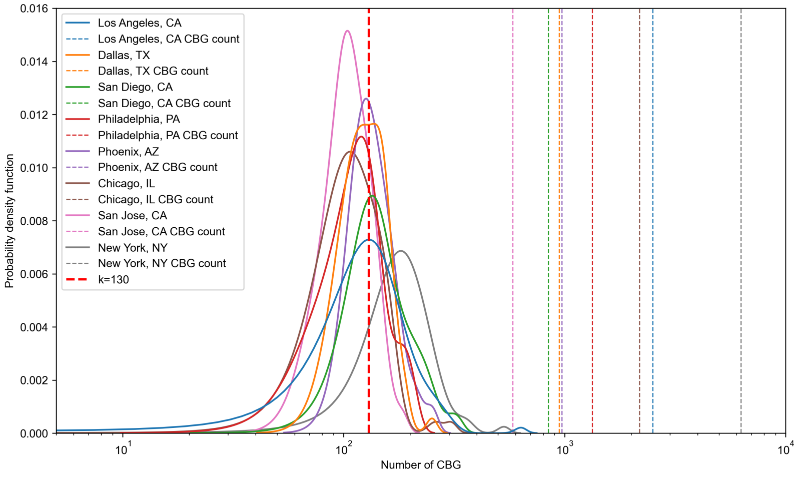

(a) The distribution of integration thresholds after removing the 2% of nodes with the smallest degrees.

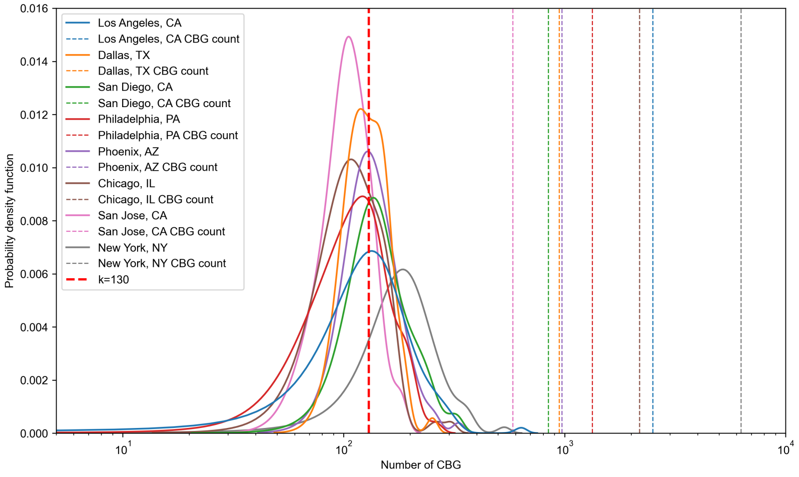

(b) The distribution of integration thresholds after removing the 3% of nodes with the smallest degrees.

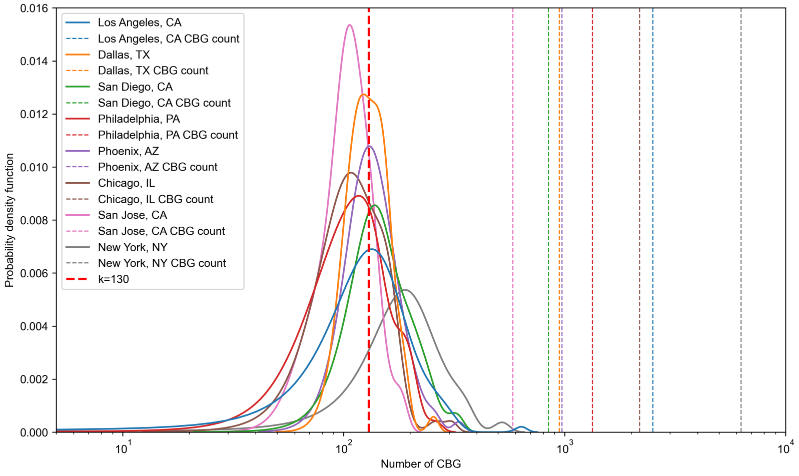

(c) The distribution of integration thresholds after removing the 5% of nodes with the smallest degrees.

FIG. S1: The Distribution of integration thresholds in different cities under three preprocessing conditions: removal of (a) 2%, (b) 3%, and (c) 5% of nodes with the smallest degrees.

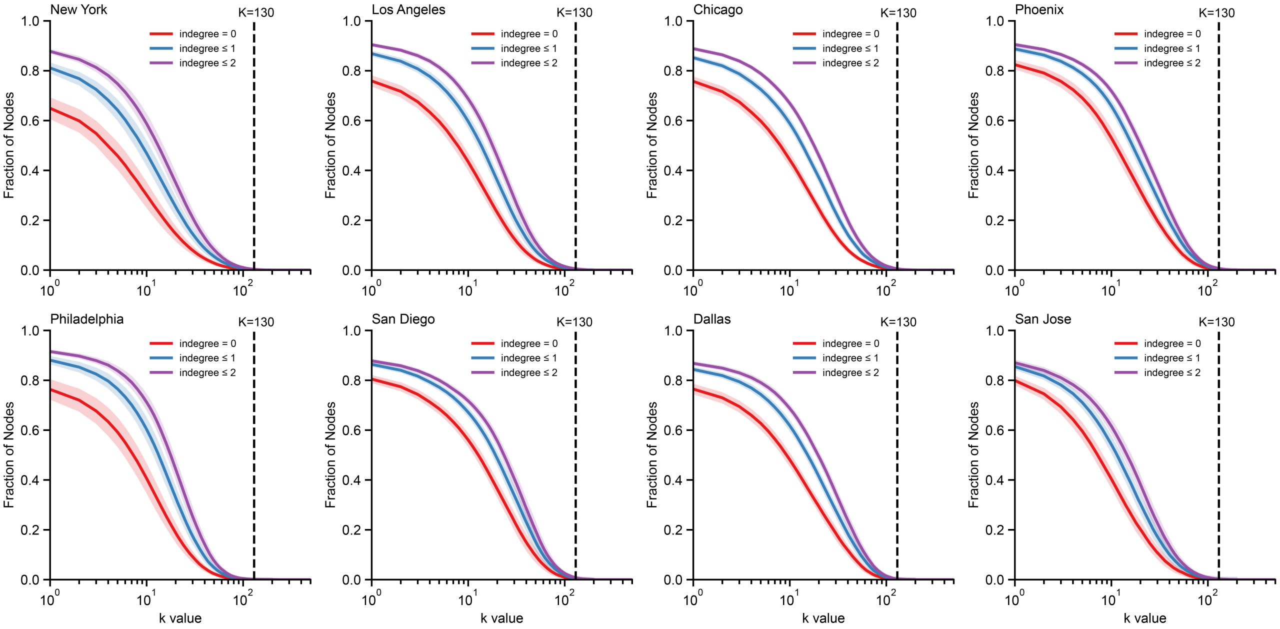

FIG. S2: Fractions of low-in-degree nodes in directed top-K mobility networks. For each city-month network, the fractions of nodes with in-degree $= 0$, in-degree ≤ 1, and in-degree ≤ 2 were computed as functions of K. Curves show monthly means for each city, and shaded bands indicate ± 1 standard deviation across monthly networks. The vertical dashed line marks the representative integration scale $K \approx 130$. Across cities, all three fractions approach zero in a narrow rank range near the representative integration scale.

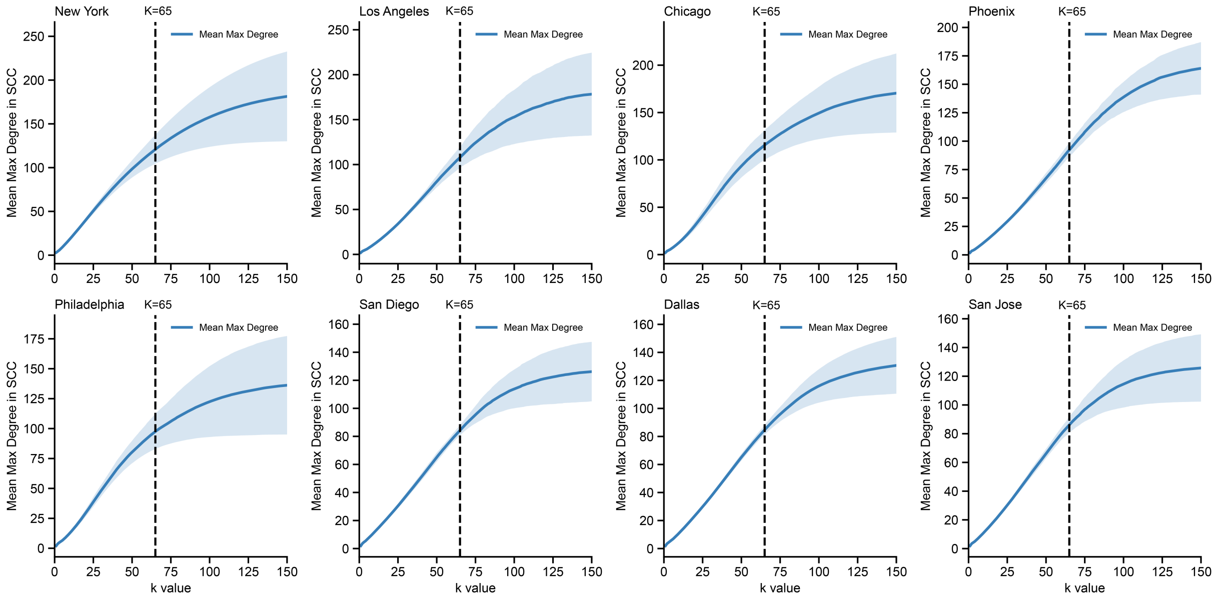

FIG. S3: Mean of the maximum in-degree across strongly connected components. For each city-month network and each value of K, the directed top- K mobility network was partitioned into strongly connected components (SCCs). Within each SCC, the maximum in-degree was identified, and the average maximum in-degree of these SCCs was then computed. Curves show monthly means for each city, and shaded bands indicate ± 1 standard deviation across monthly networks. The vertical dashed lines mark $K \approx 65$ corresponding to half of the representative scale near $K \approx 130$.

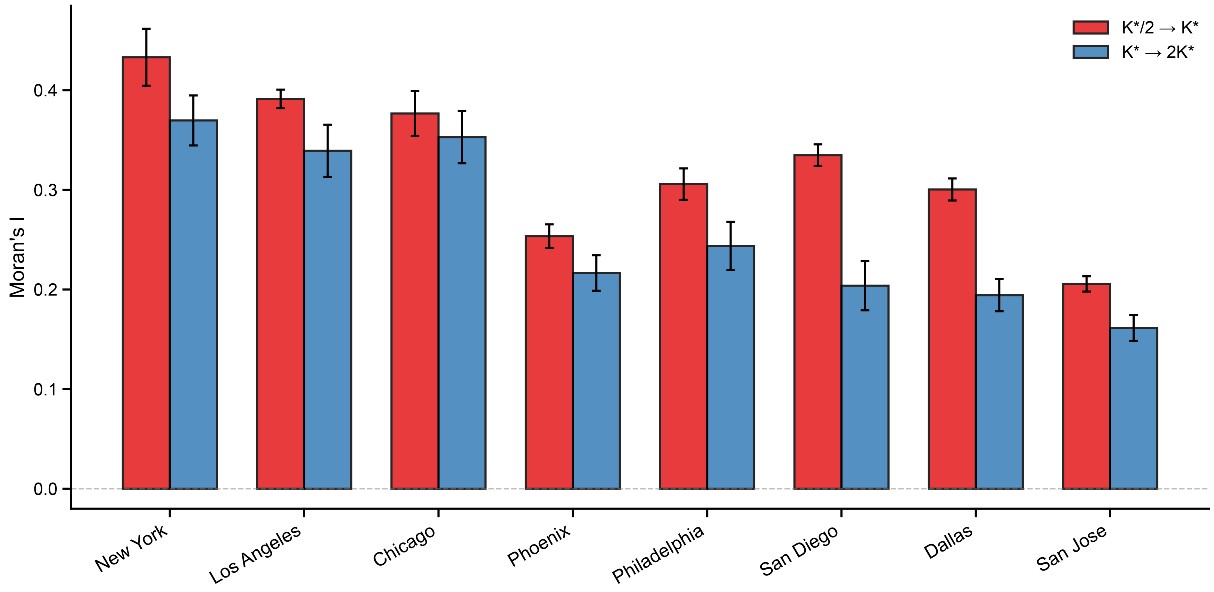

FIG. S4: Moran's I of destination frequencies across two destination-rank ranges. For each city-month network, destination frequencies were counted for the two rank ranges: $(K^*/2, K^*]$ and $(K^*, 2K^*]$. Moran's I was then computed for each range using a Queen-contiguity spatial weight matrix, in which two Census Block Groups (CBGs) were treated as neighbors if their polygons shared either a boundary segment or a vertex. Points show city-level means across months, and error bars indicate 95% confidence intervals computed as ± 1.96 standard errors. In all cities, the mean Moran's I is higher for $(K^*/2, K^*]$ than for $(K^*, 2K^*]$, and the difference is statistically significant in seven of the eight cities.

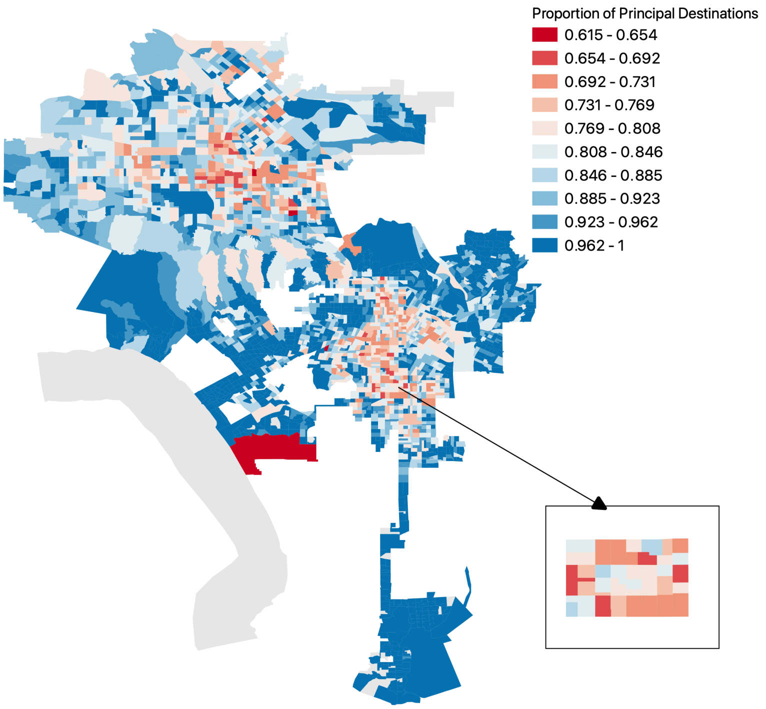

FIG. S5: Spatial Distribution of the Proportion of Principal Destinations (PPD) in Los Angeles. The inset provides a detailed view of Vermont Square, a neighborhood within the South Los Angeles region.

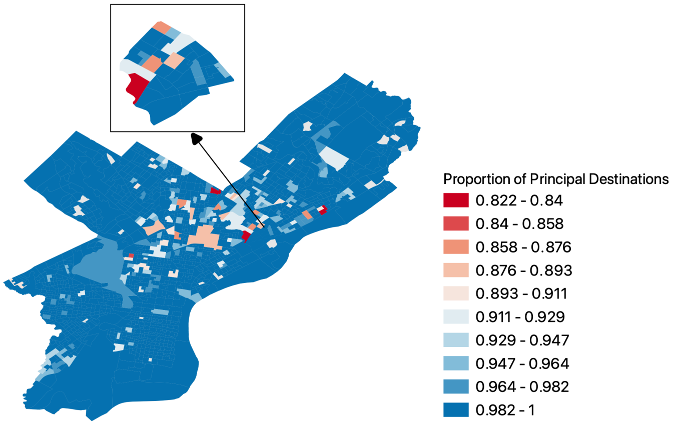

FIG. S6: Spatial Distribution of the Proportion of Principal Destinations (PPD) in Philadelphia. The inset provides a detailed view of Frankford, a neighborhood in the Northeast section of Philadelphia.

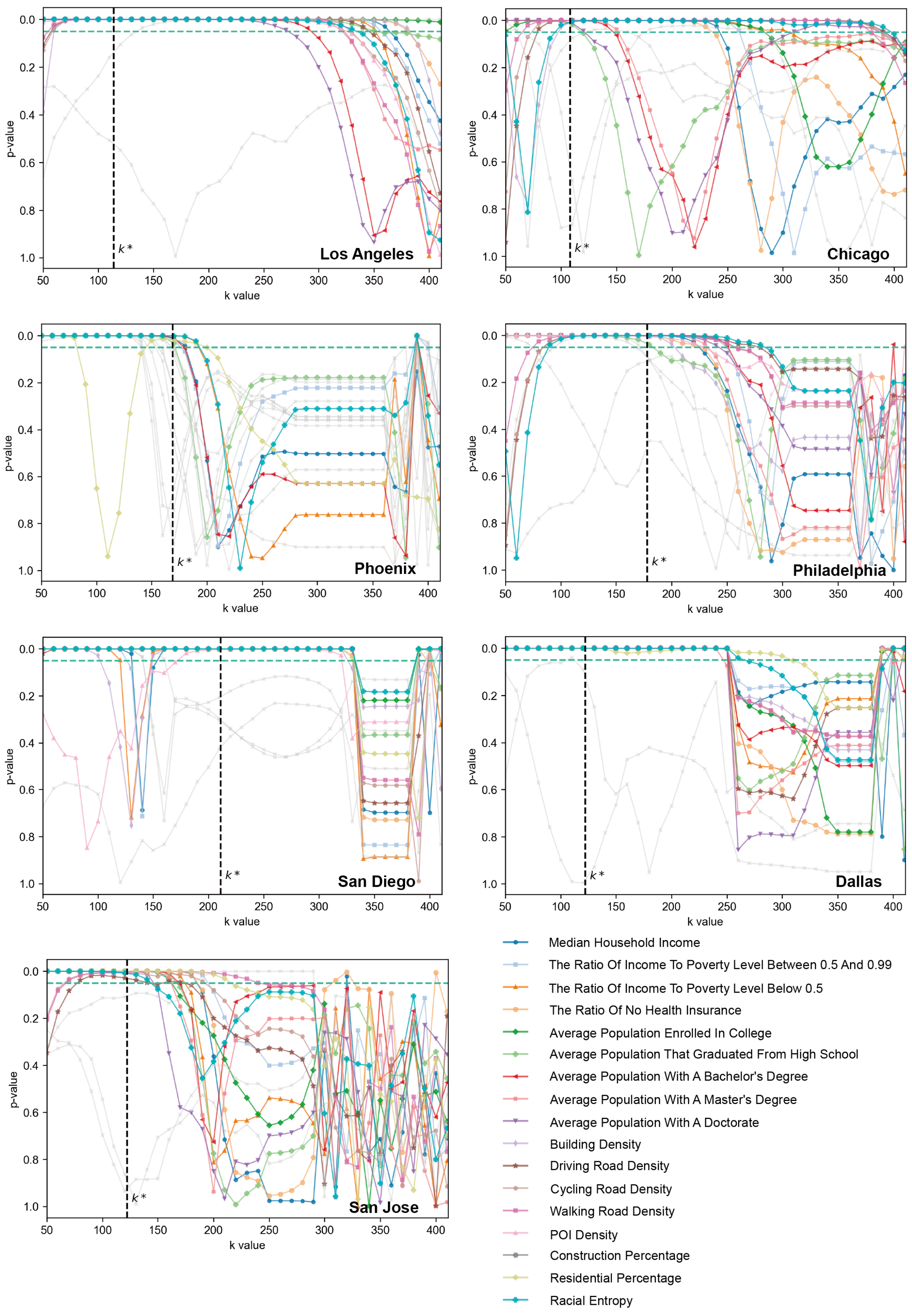

FIG. S7: The relationship between the number of principal destinations and the p-value of regressions that link socioeconomic factors with the Proportion of Principal Destinations (PPD). Vertical black dashed lines represent the integration threshold (K^*), and green horizontal dashed lines indicate a significance level of 0.05. Socioeconomic factors that show an insignificant correlation with PPD (p-value > 0.05) at the integration threshold are depicted in gray. Shifts in significance at K^* are emphasized.

[1] SafeGraph, Monthly patterns, `https://docs.safegraph.com/docs/monthly-patterns` (2022).
[2] Y. Xi, Y. Liu, T. Li, J. Ding, Y. Zhang, S. Tarkoma, Y. Li, and P. Hui, Sci. Data **10**, 866 (2023).
[3] J. M. Kleinberg, J. ACM **46**, 604 (1999).
[4] A. G. Wilson, *Entropy in Urban and Regional Modelling* (Routledge, London, 1970).
[5] F. Parés, D. G. Gasulla, A. Vilalta, J. Moreno, E. Ayguadé, J. Labarta, U. Cortés, and T. Suzumura, in *Complex Networks & Their Applications VI: Proceedings of Complex Networks 2017 (The Sixth International Conference on Complex Networks and Their Applications)* (Springer, 2018) pp. 229–240.
[6] A. Clauset, C. R. Shalizi, and M. E. J. Newman, SIAM Rev. **51**, 661 (2009).
[7] J. Alstott, E. Bullmore, and D. Plenz, PLoS One **9**, e85777 (2014).
